# Epidemiology of Maxillofacial Fractures at a Teaching Hospital in Malaysia: A Retrospective Study

**DOI:** 10.1155/2019/9024763

**Published:** 2019-02-13

**Authors:** Maher M. Abosadegh, Norkhafizah Saddki, Badr Al-Tayar, Shaifulizan Ab. Rahman

**Affiliations:** ^1^School of Dental Sciences, Universiti Sains Malaysia, Health Campus, 16150 Kubang Kerian, Kelantan, Malaysia; ^2^Hospital Universiti Sains Malaysia, Health Campus, 16150 Kubang Kerian, Kelantan, Malaysia

## Abstract

**Background/Aim:**

Epidemiology of maxillofacial fractures (MFF) varies between populations. This study investigated the epidemiology of MFF treated at the Oral and Maxillofacial Surgery (OMFS) Unit, Hospital Universiti Sains Malaysia (USM).

**Methods:**

A retrospective review of 473 medical records of patients with MFF treated from June 2013 to December 2015 was conducted. Information on demographic characteristics of patients, aetiology of injury, types of MFF, and treatment was obtained. Descriptive analysis, Pearson's chi-squared test, and multiple logistic regression analysis were conducted. The level of significance was set at 0.05.

**Results:**

Most patients treated for MFF were males (82.2%), aged 30 and below (63.1%), and from Malay ethnic (97.4%). Road traffic accident was the most common cause of MFF (83.1%), with motorcycle accident accounting for most injuries (73.6%). Orbital wall fracture was the most frequent MFF type (51.2%). About half of MFF patients (51.4%) were treated conservatively. Patients aged more than 20 years old were at higher odds of sustaining orbital wall fracture (AOR= 1.76; 95% CI: 1.214-2.558;* P*= 0.003) but were at lower odds of sustaining mandibular fracture (AOR= 0.47; 95% CI: 0.315-0.695;* P*= 0.001) than patients who are 20 years old and younger. Helmet use among motorcyclists was significantly associated with the nasal, orbital wall, and maxillary sinus wall fractures (*P=* 0.006, 0.010, and 0.004, respectively).

**Conclusion:**

Motorcycle accident was the most common cause of MFF in Kelantan, Malaysia. Ages of patient and helmet use were associated with the type of MFF sustained. This study provides important information to facilitate the planning of MFF prevention strategies among motorcyclists and emphasizes the importance of using a helmet when riding a motorcycle.

## 1. Background

Maxillofacial fractures are among the most common cause of presentations in an emergency department [1]. Maxillofacial injuries, particularly fracture, mandates special attention during diagnosis because of the close anatomical proximity to the brain and frequent association with serious concomitant injuries such as traumatic brain injury [2, 3]. The vital structures in the head and neck region must thus be evaluated whenever the maxillofacial region is suspected to be injured [3].

Maxillofacial fractures (MFF) can be considered as consequential conditions as they may result in mortality, severe morbidity, facial disfigurement, and functional limitations [4]. Early diagnosis of MFF is thus essential not only to detect concomitant injuries and emergent complications, but also to plan the reconstruction of functional areas (e.g., vision, mastication, and olfaction) and to guide physical, psychological, and social rehabilitation [5–7]. MFF can also cause considerable economic expenses due to direct procedural costs as well as indirect costs that arise from loss of productivity with associated loss of income and an inability to continue with the activities of daily life [8, 9]. Knowledge about the epidemiology of MFF can help practitioners make appropriate clinical decisions and guide the relevant professionals and policy makers develop suitable injury prevention strategies.

Epidemiology of MFF varies between populations, particularly with regard to the incidence, aetiology, and types due to environmental, socioeconomic, cultural, and lifestyle differences [10, 11]. Besides population and societal changes, the incidence and pattern of MFF may also vary between time periods due to legislative changes such as the introduction of compulsory safety belt legislation, helmet use, and speed limits enforcement [11–14]. Additionally, treatment modalities and outcomes of MFF may also vary depending on the availability, accessibility, and affordability of health care services where the study was conducted [7].

The present study determined the aetiology and types of MFF and treatment of patients seen at the Oral and Maxillofacial Surgery (OMFS) Unit, Hospital Universiti Sains Malaysia (USM), which accommodates a mix of patients from a medium-sized city and surrounding rural areas. This study also investigated the distribution of injury aetiology in relation to demographic characteristics of patients, number of MFF, and treatment modality. In addition, the association between age, helmet use, and MFF types and the factors associated with common types of MFF were determined.

## 2. Materials and Methods

We conducted a retrospective study of medical records of patients with a history of MFF treated at the OMFS Unit, Hospital USM, Kelantan, Malaysia, from June 2013 to December 2015. Located in the state capital of Kelantan, Hospital USM functions mostly as tertiary and teaching university hospital and a referral centre with a total bed capacity of 769. Kelantan state has an estimated population of 1.80 million, which includes urban and rural areas. This research was conducted in accordance with relevant ethical standards, and the study protocol was approved [USM/JEPeM/15120520] by the USM Human Research Ethics Committee [FWA Reg. No. FWA00024213; IRM Reg. No. IRB00010568] which adopts research ethics guidelines outlined by the Helsinki Declaration agreed by the World Medical Association and Council for International Organizations of Medical Sciences (CIOMS).

A total of 642 MFF cases were recorded in the registration book, and an initial review of each medical record was done to confirm the MFF diagnosis as well as to determine the eligibility of cases. The inclusion criteria were all MFF cases regardless of age, treated within the review period. The exclusion criteria were as follows: (1) patients with a history of previous MFF (repeat admission), (2) patients with existing pathological disorder involving the face such as a cyst, tumour, osteomyelitis, and fibrous dysplasia which may cause facial fractures, (3) patients with genetic disorder or congenital abnormality of the face, and (4) missing or incomplete record. Of 642 cases, 473 met the inclusion and exclusion criteria. The medical records of these 473 cases (including medical history and clinical examination notes, medical images such as X-rays and CT scans and their reports) were thoroughly reviewed.

A medical record is considered a secondary data source. Hence, the requirement for informed consent to use the patients' medical data in this study was waived by the USM Human Research Ethics Committee. However, the privacy of an individual patient was protected. All personally identifiable information such as registration number was not included in the data set, and the patients whom the data describe remain anonymous. A structured standardized proforma was designed and used to collect the variables of interest obtained from the records. These include demographic characteristics of patients (age at the time of the injury, sex, and ethnicity), aetiology of injury, type of MFF, and treatment modality. The demographic profile of patients, including the age at the time of the injury (categorised into <1, 1-10, 11-20, 21-30, 31-40, 41-50, and > 50 years), sex (male and female), and ethnicity (Malay, Chinese, Indian, and others), was also obtained. The aetiology of injury was categorised as motor vehicle accident (MVA), motorcycle accident (MCA), pedestrian, assault, fall, work-related, sports injuries, and others. The type of MFF was classified according to the following anatomical site of maxillofacial bones: alveolar palatal, nasal, naso-orbital-ethmoidal (NOE), Le Fort, maxillary sinus, zygomatic complex, zygomatic arch, orbital wall, and mandibular (subclassified into alveolar process, symphysis, body, ramus, angle, coronoid process, and condyle fractures). Treatment modality was categorised into conservative treatment (closed reduction and soft diet), surgical treatment (open reduction internal fixation), and no treatment (patient died or referred to another hospital).

The Statistical Package for Social Sciences (version 22.0, IBM, Chicago, USA) was used for data entry and analysis. The descriptive analysis was presented as frequency and percentage (%) for categorical data and mean and standard deviation (SD) for numerical data. Pearson's chi-square test was done to determine the association between age, helmet use, and MFF types with the level of significance set at 0.05. Simple logistic regression and multiple logistic regression analyses were performed to describe the association between selected independent variables (sex, age groups, ethnic group, and aetiology of injury) and the two most common types of MFF found in this study which were the orbital wall fracture and the mandibular fracture.

## 3. Results

Demographic characteristics of 473 patients treated for MFF at Hospital USM are shown in Table 1. The majority of patients were males (82.2%). The mean age of the patients was 30.6 years (SD 18.35) with a minimum age of 7 months and a maximum age of 87 years. The age group most affected by MFF was 11-20 years (34.5%) followed by 21-30 years (23.3%). Most MFF cases were Malay (97.4%), followed by Chinese, Indian, and other ethnic groups (Table 1).

Road traffic accident (RTA) was the most common cause of MFF (73.6% MCA and 9.5% MVA), followed by fall (5.7%) (Table 2). More than half of patients sustained orbital fracture (51.2%), followed by mandibular fracture (31.5%), and maxillary sinus fracture (30.7%). About half of patients who sustained MFF were treated conservatively (51.4%), while 35.7% of them underwent surgical treatment. The rest of the patients did not have any active treatment done at Hospital USM and were either referred or died.

Table 3 summarizes the distribution of various MFF aetiologies with regard to demographic characteristics of patients, number of MFF, and treatment modalities. The most common aetiology of injury in both male and female patients was MCA, followed by MVA. Similarly, MCA and MVA were the common aetiologies in patients aged ≤20 years and >20 years. Assault and fall were more common in males than in females and were more common among patients aged >20 years than in younger patients.

MCA was also the most common injury aetiology among the Malay ethnic group (74.6%), followed by MVA (8.5%) and assault (5.6%), while in other ethnic groups MVA was the most common (50.0%). MCA was the most common MFF aetiology regardless of the number of fractures sustained by the patients and the type of treatment rendered.

The associations between age group and MFF types are shown in Table 4. In general, age group of patients was significantly associated with a fracture to the following maxillofacial bones: zygomatic complex, zygomatic arch, orbital wall, and mandible (P= 0.001, 0.004, 0.002 and <0.001, respectively). Most patients who sustained zygomatic complex, zygomatic arch, and orbital wall fractures aged more than 20 years (74.1%, 74.4%, and 66.9%, respectively). On the other hand, the mandibular fracture affected younger (aged 20 years old and below) (52.3%) than older patients (above 20 years old) (47.7%).

Of 348 patients who sustained MFF during MCA, only 142 (40.8%) used helmets while others did not.  Table 5 shows the association between helmet use and MFF types among motorcyclists. Helmet use among motorcyclists was significantly associated with nasal, orbital wall, and maxillary sinus wall fractures (*P*= 0.006, 0.010, and 0.004, respectively).

Table 6 presents results of simple and multiple logistic regression analyses of factors associated with orbital wall fracture, the most common MFF type among patients treated at Hospital USM. Age was significantly associated with the incidence of orbital wall fracture at both univariable and multivariable levels. Patients aged more than 20 years old were at higher odds of sustaining orbital wall fracture than patients who were 20 years old and younger with a crude and adjusted odds ratio (OR) of 1.78 (95% CI:1.23-2.58) and 1.76 (95% CI: 1.21-2.56), respectively. The influence of other variables was not statistically significant.

Table 7 shows the results of simple and multiple logistic regression analyses of factors associated with mandibular fracture, the second most common MFF type among patients treated at Hospital USM. Age was also the only variable found to be significantly associated (albeit inverse) with the incidence of mandibular fracture at both univariable and multivariable levels. Patients aged more than 20 years old were at lower odds of sustaining a mandibular fracture than patients who were 20 years old and younger with crude and adjusted OR of 0.47 (95% CI: 0.32-0.70). The influence of other variables was not statistically significant.

## 4. Discussion

MFF not only cause serious injuries to the victim but also impose a serious burden on the society due to morbidity, mortality, facial disfigurement, loss of function, and financial expenses associated with the injuries [8, 9]. The incidence, aetiology, types, and associated injuries of MFF vary between different countries and even different areas of the same country due to environmental, socioeconomic, cultural, and lifestyle differences among people [10–12]. This study analyzed 473 MFF cases treated at the OMFS Unit, Hospital USM, Kelantan, Malaysia.

The ethnicity of patients treated for MFF in this study was generally similar to the composition of Kelantan population [15], with most participants being of Malay ethnic, followed by Chinese, Indians, and other groups. The proportion of males affected by MFF in this study was higher than females, in agreement with findings reported in most other studies [10, 16–20]. The male preponderance in this study is not surprising as males were reported to be about 6.5 times more likely than the females to sustain major trauma in Malaysia, 86.6% males versus 13.4% females [21]. The local lifestyle and culture also, to a certain extent, still confine men and women to their traditional social roles. Men have a more active presence in the society and work outside the home, while women are restricted to the domestic sphere, and if they do work, it is mainly office work or another stereotypically female profession [20, 22]. Statistics on women, family, and community published by the Ministry of Women, Family and Community Development Malaysia in 2016 showed that labour force participation rate has always been higher in males than females [23]. As there are more men than women working, there will be more men than women commuting. This explains the higher occurrence of RTA among males than females as reported in previous studies [24–26]. Our results also showed that while RTA was the most common cause of injury in both male and female patients, more males than females were involved in RTA.

RTA was found to be the most common aetiology of MFF in this study. This is in agreement with findings from studies done in other countries such as India, Iran, Greece, Japan, Netherland, and Tanzania [5, 18, 27–30]. In Victoria, Australia, while RTA was found to be the most common cause of MFF in a study on data obtained from 2001 to 2004 [16], another retrospective study on cases managed at the Alfred Hospital, also in Victoria from 2009 to 2011, showed that assault was the most common mechanism of injury [31]. Another recent multicentric retrospective study from Southern Italy in 2018 showed that the most frequent cause of facial injuries was assault (30.4%), followed by RTA (27.2%), and falls (23.2%) [32]. In contrast to that, the study done by Bocchialini et al. from Brescia, Italy, reported RTA as the main cause of MFF (20%), followed by assault (14.4%), and sports (14.1%) [12]. These findings highlighted the variation in the epidemiology of MFF which changes over time even in a similar area of the same country.

In this study, most of MFF was due to RTA involved motorcycles. A study in Penang Mainland, Malaysia, also showed that MCA was the main cause of MFF [33]. This is probably because a motorcycle is one of the major modes of personal transport in Malaysia [34]. Small- and medium-sized motorcycles (engine sizes of 150 cubic centimetres (cc) and below) are convenient and affordable with the low purchase price and insurance rates, and the motorcycle licenses can be obtained at the minimum age of 16 years old. Similarly, MCA accounted for the majority of MFF in India, Greece, Japan, and Tanzania [5, 20, 27, 29, 30], while car accidents were more common in Australia [16] and bicycle accidents were more common in the Netherlands [28]. In Iran, car accidents were found to be the common cause between the period from 1996 to 2001 [17]. However, between 2012 and 2014, MFF in Iran were mostly caused by MCA [18].

In this study, almost half of MCA victims were below the age of 21 years (46.0%). This is in agreement with recent findings of a 10-year study regarding the incidence and etiology of MFF done by Pungrasmi and Haetanurak in Thailand which report MCA as the most common cause of MFF and half of patients were 11-30 years old [35]. This is possibly because the young motorcyclists are inexperienced, lack the proper riding skills, often ride carelessly or recklessly, and tend to violate traffic laws, for example, by not using helmets, running red lights, and exceeding the speed limit [20, 36, 37]. Besides RTA, fall, assault, and pedestrian accidents were also the common causes of MFF in this study, in agreement with the reports of other studies [5, 28, 29].

The most common MFF site or type following trauma varies between studies. Results from most studies have shown that mandible was most commonly affected [5, 17, 18, 27–30, 38]. However, in this study, the orbital wall was found to be the most common injury site (51.2%), followed by mandible (31.5%) and maxillary sinus (30.7%). Orbit was also the most common site of MFF reported in an Australian study [31]. However, in another Australian study, maxilla was found to be the most frequent maxillofacial bone affected in major trauma (22.3%), followed by orbit (21.4%), while mandible was less commonly injured (9.6%) [16]. This variation in MFF types following trauma can be explained by differences in the mechanism of injury, magnitude and direction of impact force, and anatomy of the site. In the Australian studies, maxilla was most prevalent where RTA was the most common cause of injury [16], while orbital fracture was most prevalent where the assault was the most common mechanism [31]. RTA was also reported to be the most common cause of fractures of the mandible [39].

MFF can occur at any age. In this study, most MFF were seen in young adults. Similar findings have been reported in other studies [5, 10, 16, 18, 27–29, 31, 38]. Higher susceptibility of this age group to MFF may be due to their involvement in sports and other physical activities or in psychosocial problems that may potentiate risk-taking behaviors, hence making them more prone to injuries [40]. In this study, the age of patients was found to be associated with sites of fracture. Looking at the two most common types of MFF in this study, it was demonstrated that patients aged 20 years or older were more likely to have orbital wall fracture but less likely to sustain mandibular fracture following trauma. A retrospective review by Atisha et al. also showed that elderly patients sustained a higher incidence of orbital floor fractures and a lower incidence of mandible fractures than younger patients [41]. In agreement, mandibular fracture accounted for most facial fractures encountered in young patients as opposed to the midface which is mainly protected due to its retrusive position relative to the prominent calvaria [42]. The mandible is more vulnerable and weak in young age, especially those at the age of having mixed dentition, due to the presence of tooth buds in the bone. On the other hand, the midface and zygomatic bone are less vulnerable in young age as the anatomical structure is not fully developed such as maxillary antrum and the orbital socket.

In view that MCA was the most common cause of MFF in this study, the most important preventive measure is to practice safe riding to avoid crashes. Nevertheless, MCA can occur despite the best prevention efforts, and sometimes it is not the motorcyclist's fault. Hence, the use of personal protective gear such as leather jackets, gloves, trousers, appropriate footwear, eye protection, and helmets is important to reduce injury. Of these, helmets are the most important safety equipment because they protect not only against injuries to the head and brain but also against fractures of the maxillofacial bones [13] which in turn may increase the risk for head and brain injuries [2, 3].

In the present study, patients who wore a helmet had a significantly lower incidence of nasal fracture, orbital wall fracture, and maxillary sinus wall fracture than those who did not wear a helmet. Fractures to other maxillofacial bones were also lower in patients who wore helmets in this study although the differences were not significant. Similarly, findings of a study by Christian et al. to investigate the difference in incidence and pattern of injuries sustained by helmeted versus nonhelmeted motorcyclists in Memphis, United States, showed that helmeted patients recorded significantly lower incidence of facial injury and fracture than nonhelmeted patients, particularly incidence to the orbit and maxilla [43]. A study in Kerala, India, among patients attending the emergency department showed that the incidence of facial bone fractures was significantly higher in nonhelmeted than helmeted individuals, 53% and 14%, respectively, further substantiating the protective role of the helmet in preventing MFF [13].

MFF can be treated with either closed reduction (conservative) or open reduction and internal fixation (ORIF) (surgical) methods, or a combination of approaches. The decision regarding treatment depends upon a variety of factors such as nature of the injury, the presence of associated injuries, and comorbidities, skills of the surgeon, availability of facilities and instruments, and patient's ability to pay for the treatment cost [5]. In this study, more than half of MFF cases were treated conservatively.

This study investigated the epidemiology of MFF and the associated risk factors among patients treated at Hospital USM, Kelantan, Malaysia, which is one of the largest tertiary referral hospitals in the state capital of Kelantan and functions mostly as teaching hospital and a referral centre for all cases including the cases that require more complex management from both rural and urban areas. However, the retrospective nature of this study has inherent limitations due to incomplete records, gap information, and information obtained based on assessment and documentation by various medical professionals.

## 5. Conclusion

RTAs, particularly MCA, were the most common cause of MFF in Kelantan, Malaysia. MFF injury site was associated with age of patient and helmet use. This study provides useful data for planning prevention strategies of MFF, particularly among motorcyclists, and emphasizes the importance of using personal protective gear as helmets when riding a motorcycle.

## Figures and Tables

**Table 1:** Demographic characteristics of patients with MFF treated at Hospital USM (n=473).

Variable	Frequency (%)
*Sex*	
Male	389 (82.2)
Female	84 (17.8)
*Age group (year)*	
< 1	1 (0.2)
1-10	24 (5.1)
11-20	163 (34.5)
21-30	110 (23.3)
31-40	39 (8.2)
41-50	54 (11.4)
> 50	82 (17.3)
*Ethnic group*	
Malay	461 (97.4)
Chinese	6 (1.3)
Indian	1 (0.2)
Others	5 (1.1)

**Table 2:** Aetiology, type, and treatment of patients with MFF at Hospital USM, (n=473).

Variable	Frequency (%)
*Aetiology of MFF*	
MCA	348 (73.6)
MVA	45 (9.5)
Pedestrian	17 (3.6)
Assault	17 (3.6)
Fall	27 (5.7)
Work-related	7 (1.5)
Sport	6 (1.3)
Other	6 (1.3)
*Type of MFF*	
Alveolar palatal	56 (11.8)
Nasal	125 (26.4)
NOE	28 (5.9)
Le Fort	102 (21.6)
Maxillary sinus	145 (30.7)
Zygomatic complex	108 (22.8)
Zygomatic arch	82 (17.3)
Orbital wall	242 (51.2)
Mandibular	149 (31.5)
Alveolar process	49 (10.4)
Symphysis	101 (21.4)
Ramus	7 (1.5)
Angle	34 (7.2)
Body	22 (4.7)
Coronoid process	8 (1.7)
Condyle	74 (15.6)
*Treatment modality*	
Conservative treatment	243 (51.4)
Surgical treatment	169 (35.7)
No treatment	61 (12.8)

MCA, motorcycle accident; MVA, motor vehicle accident; NOE, naso-orbital-ethmoidal.

**Table 3:** Aetiology of MFF with reference to demographic characteristics of patients, number of MFF, and treatment modality (n=473).

Variable	Aetiology of MFF [Frequency(%)]								Total (%)
	MCA	MVA	Pedestrian	Assault	Fall	Work-related	Sport	Other	
*Sex*									
Male	289 (74.0)	32 (8.2)	10 (3.0)	15 (4.0)	24 (6.0)	7 (1.8)	6 (1.5)	6 (1.5)	389 (82.2)
Female	59 (70.2)	13 (15.5)	7 (8.3)	2 (2.4)	3 (3.6)	0 (0.0)	0 (0.0)	0 (0.0)	84 (17.3)
*Age group (year)*									
≤20	160 (85.1)	10 (5.3)	2 (1.1)	5 (2.7)	6 (3.2)	0 (0.0)	4 (2.1)	1 (0.5)	188 (39.7)
>20	188 (66.0)	35 (12.3)	15 (5.3)	12 (4.0)	21 (7.4)	7 (2.5)	2 (0.7)	5 (1.8)	285 (60.3)
*Ethnic group*									
Malay	344 (74.6)	39 (8.5)	17 (3.7)	17 (3.7)	26 (5.6)	7 (1.5)	5 (1.1)	6 (1.3)	461 (97.5)
Others	4 (33.4)	6 (50.0)	0 (0.0)	0 (0.0)	1 (8.3)	0 (0.0)	1 (8.3)	0 (0.0)	12 (2.5)
*Number of MFF per patient*									
1	146 (73.4)	11 (5.5)	7 (3.5)	11 (5.5)	14 (7.1)	2 (1.0)	6 (3.0)	2 (1.0)	199 (42.0)
2	66 (69.5)	11 (11.6)	5 (5.3)	3 (3.0)	6 (6.3)	1 (1.1)	0 (0.0)	3 (3.2)	95 (20.1)
3	49 (71.0)	9 (13.0)	2 (3.0)	2 (2.9)	5 (7.2)	2 (2.9)	0 (0.0)	0 (0.0)	69 (14.6)
≥ 4	87 (79.2)	14 (12.7)	3 (2.7)	1 (0.9)	2 (1.8)	2 (1.8)	0 (0.0)	1 (0.9)	110 (23.3)
*Treatment modality*									
Conservative treatment	176 (72.0)	15 (6.2)	11 (5.0)	14 (5.8)	16 (7.0)	3 (1.0)	3 (1.0)	5 (2.0)	243 (51.4)
Surgical treatment	127 (75.0)	21 (12.4)	4 (2.4)	3 (1.8)	6 (3.6)	4 (2.4)	3 (1.8)	1 (0.6)	169 (35.7)
No treatment	45 (73.8)	9 (14.8)	2 (3.3)	0 (0.0)	5 (8.2)	0 (0.0)	0 (0.0)	0 (0.0)	61 (12.9)

**Table 4:** Association between age group and types of MFF (n=473).

Variable	Frequency(%)		*P*-value**∗**
	≤20 years	>20 years	
*Alveolar-palatal*			
** **No	170 (40.8)	247 (59.2)	0.216
** **Yes	18 (32.1)	38 (67.9)	
*Nasal*			
** **No	140 (40.2)	208 (59.8)	0.720
** **Yes	48 (38.4)	77 (61.6)	
*NOE*			
** **No	178 (40.0)	267 (60.0)	0.653
** **Yes	10 (35.7)	18 (64.3)	
*Le Fort*			
** **No	155 (41.8)	216 (58.2)	0.085
** **Yes	33 (32.4)	69 (67.6)	
*Zygomatic complex*			
** **No	160 (43.8)	205 (56.2)	**0.001**
** **Yes	28 (25.9)	80 (74.1)	
*Zygomatic arch*			
** **No	167 (42.7)	224 (57.3)	**0.004**
** **Yes	21 (25.6)	61 (74.4)	
*Orbital wall*			
** **No	108 (46.8)	123 (53.2)	**0.002**
** **Yes	80 (33.1)	162 (66.9)	
*Maxillary sinus wall*			
** **No	138 (42.1)	190 (57.9)	0.120
** **Yes	50 (34.5)	95 (65.5)	
*Mandible*			
** **No	110 (34.0)	214 (66.0)	**<0.001**
** **Yes	78 (52.3)	71 (47.7)	

*∗*Chi-square test.

**Table 5:** Association between helmet use and MFF types among motorcyclists (n=348).

Variable	Frequency(%)		*P*-value*∗*
	Used helmet (n=142)	Did not use helmet (n=206)	
*Alveolar-palatal*			
No	126 (41.0)	181 (59.0)	0.805
Yes	16 (39.0)	25 (61.0)	
*Nasal*			
No	116 (45.1)	141 (54.9)	**0.006**
Yes	26 (28.6)	65 (71.4)	
*NOE*			
No	131 (40.3)	194 (59.7)	0.478
Yes	11 (47.8)	12 (52.2)	
*Le Fort*			
No	108 (40.0)	162 (60.0)	0.570
Yes	34 (43.6)	44 (56.4)	
*Zygomatic complex*			
No	112 (41.0)	161 (59.0)	0.873
Yes	30 (40.0)	45 (60.0)	
*Zygomatic arch*			
No	121 (42.3)	165 (57.7)	0.220
Yes	21 (33.9)	41 (66.1)	
*Orbital wall*			
No	82 (47.7)	90 (52.3)	**0.010**
Yes	60 (34.1)	116 (65.9)	
*Maxillary sinus wall*			
No	111 (45.9)	131 (54.1)	**0.004**
Yes	31 (29.2)	75 (70.8)	
*Mandible*			
No	87 (37.8)	143 (62.2)	0.114
Yes	55 (46.6)	63 (53.4)	

*∗*Chi-square test.

**Table 6:** Factors associated with orbital wall fracture among patients using multiple logistic regression analyses.

Variable	Crude OR^a^ (95% CI)	*P*-value^a^	Adjusted OR^b^ (95% CI)	*P*-value^b^
*Gender*				
Female	1.00			
Male	1.34 (0.83-2.14)	0.232		
*Age group (year)*				
≤20 years	1.00			
>20 years	1.78 (1.23-2.58)	0.002	1.76 (1.21-2.56)	**0.003**
*Ethnic groups*				
Others	1.00			
Malay	0.74 (0.23-2.37)	0.616	-	-
*Aetiology of MFF*				
Other	1.00			
Fall	0.76 (0.38-1.50)	0.681	-	-
RTA	1.18 (0.54-2.58)	0.420	-	-

^a^Simple logistic regression.

^b^Multiple logistic regression.

**Table 7:** Factors associated with mandible fracture among patients using simple and multiple logistic regression analyses.

Variable	Crude OR^a^ (95% CI)	*P*-value^a^	Adjusted OR^b^ (95% CI)	*P*-value^b^
*Gender*				
Female	1.00			
Male	0.85 (0.51-1.39)	0.511	-	-
*Age group (year)*				
≤20 years	1.00			
>20 years	0.47 (0.32-0.70)	0.001	0.47 (0.32-0.70)	**0.001**
*Ethnic groups*				
Others	1.00		-	
Malay	0.43 (0.09-1.97)	0.420		
*Aetiology of MFF*				
Other	1.00			
Fall	0.68 (0.20-2.33)	0.542	-	-
RTA	1.47 (0.67-3.22)	0.332	-	

^a^Simple logistic regression.

^b^Multiple logistic regression.

## Data Availability

The data used to support the findings of this study are available from the corresponding author upon request.
